# Supplementary material for: Resting-state background features demonstrate multidien cycles in long-term EEG device recordings
Source: Brain Stimul. Author manuscript; Available in PMC 2026 Mar 5. (PMC12961971; doi:10.1016/j.brs.2023.11.005)
Supplement: Supplementary Material [file NIHMS2142166-supplement-Supplementary_Material.docx]

**Supplementary Information for “Multidien cycles in interictal background features of long-term EEG recordings”**

William K.S. Ojemann*, Brittany H. Scheid*, Sofia Mouchtaris, Alfredo Lucas, Joshua J. LaRoque, Carlos Aguila, Arian Ashourvan, Lorenzo Caciagli, Erin C. Conrad, Brian Litt

**Contents**

1. Supplementary Methods
2. Supplementary Results
3. Supplementary Figures
4. Supplementary Tables

## Supplementary Methods

###

### Patient Selection

We excluded eight patients for the following reasons: insufficient IEA detection data (N=2), lack of a dominant multidien cycle (N = 3), insufficient scheduled EEG recording clips ( < 200) without stimulation (N=4), insufficient scheduled EEG recording clips with stimulation (N = 6, 3 patient exclusion overlap) (**Figure S4**). Patients without a significant, dominant dIEA cycle were excluded from analysis because the peaks in their multiday power spectrum, while measurable, were indistinguishable from spectral peaks driven by random oscillations in the data (e.g. noise).

### dIEA Cycle Analysis

We first removed the first 30 days of dIEA data to account for potential implant effects. We then normalized the hourly dIEA counts between neurologist visits to account for detection parameter changes (**Figure 1A**). Data gaps of 5 hours or less were linearly interpolated, otherwise data was segmented at larger recording gaps. We applied a Morlet wavelet decomposition to each continuously recorded segment to obtain a spectrogram (octaves: 12, minimum scale: 0.008). We averaged the spectrogram across time to obtain a time-averaged periodogram, then averaged periodograms across segments to obtain a single periodogram per patient (**Figure 1B**). We identified peaks in the time-averaged periodogram using MATLAB’s *findpeaks* function. Periods corresponding to peaks that rose above the 99% confidence interval of the Fourier red-noise null model were identified as significant dominant modes of the dIEA count signal [1], [2].

For each dominant mode in the range of 3-60 days, we applied the inverse wavelet transform to reconstruct the component of the signal with the associated period (basis function: delta function, reconstruction factor: 0.776, decorrelation factor: 2.32, scale averaging factor: 0.60, energy de-scaling factor: -1/4) [3]. Prior studies report that significant multidien cycles are not present in all subjects [4], [5] and five patients did not have a significant dominant mode within the 3-60 day window and were excluded from the study. For the remainder of our analysis, we selected the multidien period in each patient that corresponded most with seizure occurrence. That is, we used Long Episodes (LE) as a proxy for seizures and chose the multidien period with the highest phase-locking value of LE to the reconstructed (inverse wavelet) signal [4], [6] (**Figure 1B,C**). Finally, we used the Hilbert transform to obtain the instantaneous phase angle for each timepoint of the chosen reconstructed hourly dIEA cycle (**Figure 1C**).

### Spike Detection

We built our spike detector with the following settings: data in each SE clip was filtered between 10 and 100 Hz using a 6th order butterworth bandpass filter. The channel-specific peak-to-peak spike amplitude threshold criteria was defined as the maximum of 7.5 standard deviations above the mean across all SE clips or 6.5 times the absolute median of the data within the clip. Setting dual thresholds ensured that the detector was not overly sensitive when clip amplitude was elevated. Additionally, the maximum and minimum spike peaks were required to fall within a window of 15 to 200 ms (**Figure 2A**). Any spikes that occurred within 1 second of a stimulation interval were excluded due to stimulation artifacts.

### Feature Calculation

To calculate band-limited bandpower in each frequency band of interest (theta, alpha, beta, gamma) we applied the MATLAB *bandpower* function to the entirety of the recording window - either an entire scheduled event without stimulations or the window after stimulation. We then took the maximum band power value between the two detection channels in each frequency band to get one band power feature value per recording, per frequency. We excluded the delta frequency band due to onboard filtering settings in the RNS device that prevent analysis of this activity.

To calculate band-limited connectivity we first split the recording of interest into non-overlapping one second clips. We then, in each clip, calculated the cross-wavelet transform connectivity using the MATLAB function *xwt* [7] between the two recording channels on the same electrode in each frequency band of interest providing one connectivity value per clip, per electrode, per frequency band. We then averaged across all of the clips to obtain an average connectivity value for each electrode in each frequency band and, in each frequency band, took the maximum value between the two electrodes. This provided one connectivity value per frequency band in each recording.

An important note in interpreting our EEG-dIEA results is that, while dIEA detection criteria can include thresholds of bandpower ranges as well as some spikes, our analysis investigates background signals explicitly when these detection criteria were not met - windows after detections or recordings without any detections.

In our initial exploration of feature values, we observed a significant drift in values across different programming intervals, such that any cyclic variation on a 7-30 day period would be obscured by larger shifts in the feature baseline. The drifts could be due to programming parameter changes, which could adjust the absolute amount of simulations per day and could affect the baseline level of activity, changes in medication, which typically occur near programming visits, or other changes in device recording hardware, or long term brain plasticity. To address changes in detection parameters and baseline drifts in the features, we z-scored each feature between clinical neurologist visits.

### Machine learning modeling

During univariate feature analysis, we observed a high correlation between feature sets (**Figure S8**), and thus applied a principal component analysis (PCA) transformation to the feature data in each fold to mitigate the multicollinearity [8]. Only the first n principal components that explained 95% of the variance became our training features, in order to limit the destabilizing effect of principal components with smaller associated eigenvalues. We weighted the cost of misclassification by initializing our models using the class imbalance of the training fold, to prevent training-class imbalance from affecting our model generalizability. All predictions that we generated were out-of-sample predictions on the validation dataset for each fold, meaning that their accuracy and effect size is a better representation of how accurately the model is learning the relationship between the features and response variable.

To interpret the relationship between the features and response variable, we deployed an extension of principal regression analysis and applied the inverse PCA transform to the standardized model coefficients - scaled by the variance of the explanatory and response variables in each fold - to transform them back into feature space and compare across patients [8]. Because we use a 5-fold cross-validation paradigm to assess the performance of our models, to get one feature importance value per patient-specific model we take the average feature value across k-folds. Analysis of the patient-specific model coefficients (**Figure S9**) reveals that coefficients are highly consistent across k-folds. When we plot the distribution of model coefficients for both the stimulation-free and post-stimulation recordings we only plot the coefficients of the patients who had a validation AUC above 0.5. The reason for this exclusion is to prevent any coefficient inflation due to overfitting from biasing the population coefficient distributions.

To assess the significance of each model that we trained (PvT, RvF, PvR, RvT, multivariate) and account for any potential AUC inflation due to class imbalance we built a null distribution of 1000 null models. We generated the null models using the following protocol: For each permutation, we randomly permuted the label assignments (e.g. peak/trough) across the dataset, and then replicated our 10-fold cross validation training paradigm. We then stored the average AUC across the 10-folds for each permutation to generate an N=1000 null AUC distribution. To assess the significance of the model trained on the original dataset we derived from the SE recordings, we performed a one-sided permutation test:

$$p_{permutation}=\frac{\sum_{n=1}^{N} I(AUC<AUC_{perm,n})+1}{N + 1}$$

Where $I$ is the indicator function, and we add 1 to the numerator and denominator to avoid the theoretically impossible p-value of 0 [9].

## Supplementary Results

### Scheduled event distribution

In addition to performing group level analyses examining the distribution of scheduled events across dIEA cycle phases, we also performed the same analysis at the individual level to assess the effect in single patients. Class imbalance was assessed by a chi-squared goodness of fit test to a uniform distribution. We observed 7/19 patients had a significant class imbalance in the distribution of their recordings without stimulation (**Figure S3A**). We observed 12/19 patients had a significant class imbalance in the distribution of their recordings containing stimulation **(Figure S3B**).

We would expect overall a smaller number of SE clips recorded during rising and peak phases of the cycle, given the phase dependence of LEs and increased possibility of overwritten SEs. However, when looking at clips with stimulation alone, the bias for there being more SEs with stims during rising and peak phases appears to counteract the bias due to overwriting, thus resulting in the opposite distribution of relative counts.

Because the highly irregular sampling of SEs compared to dIEA limited our ability to apply circular analyses and statistics to the background features we extracted, we show the inter-recording interval for an example patient (**Figure S3C**). This plot includes both SEs with stimulation and without stimulation, such that each of those groups individually would be even more fragmented.

### dIEA features sub-analyses

In addition to using dIEA cycles to study long-term fluctuations in background features, we also further explored the relationship between features of the dIEA signal. Examining the distribution of LE phase-locking to dIEA signals reveals that our patient population is similar to previously reported results (Leguia et al., 2021) (**Figure S2A**). We also examine the spearman rank correlation between different features extracted from the dIEA signal, as well as between those features and our binary model performance. When examining the relationship between the peak period that had the highest LE-PLV and the length of the period, we observed a significant negative correlation between the two variables (**Figure S2B**). After generating models defining the association between background connectivity and bandpower features, and dIEA cycle phase bins we were interested in identifying covariates that could explain the variance in model effect size. We observed no significant correlations between PvT and RvT AUC and LE-PLV or dIEA cycle period length (**Figure S2C**). This trend persists in models that have spikes as a covariate as well, with no significant correlations. We were also interested in understanding how seizure frequency might affect the performance of our background feature models. To understand this relationship, we measured the spearman rank correlation between baseline seizure frequency in seizures per week and PvT model AUC. We observe no significant association between the two features (**Figure S2D**).

### Additional univariate analyses

The first analysis we performed when examining the relationship between features and dIEA phase was to compare values across all four phase groups to test for any differences. The results of this ANOVA are shown in **Figure S6** where all significant comparisons are colored, and interpreted in the main manuscript. In addition to examining the patient specific univariate feature PvT effect sizes (**Figure 3B**), we also include the patient specific univariate RvF effect sizes, and the distributions across the patient population for both phase comparisons (**Figure S6**). The population level feature distributions reveal significant trends in theta connectivity, beta connectivity, theta band power, and alpha band power.

### RNS detection criteria and multiday cycles

Onboard detectors track signal features (e.g. signal band power, line length, zero crossings) in up to two channels at a time, and are triggered when the feature exceeds a preset threshold over a specified time interval. Detectors are tuned by a licensed neurologist to be triggered by both short epileptiform discharges, if the detection threshold is exceeded briefly (usually < 2s), and by seizures, if the detection threshold is exceeded for a longer period of time (usually 10-30s). Device detection parameters vary across patients and could underlie the variation in model performance. In order to understand whether patient-specific differences in model performance could be driven by individualized detention parameters, we show the most stable detection parameters settings for each patient in **Figure S12**, which demonstrates both the variety and considerable overlap of detection parameters across patients selected for our analysis. All but four subjects (HUP059, HUP108, HUP129, HUP137) had detection parameters that collectively spanned the frequency bands of interest. And in those four subjects whose detection parameters were constrained to lower frequency bands, of the two who were included in our background model analysis (HUP129 & HUP137) **Figure S9**, it was not obvious that the lower frequency features were driving model performance. Therefore, while we cannot be certain that individual detection settings did not contribute to the variance between subjects, we did not observe a measurable bias in a patient group with a different range of detection settings.

### Anatomical location subgroup analyses

We were initially motivated to better understand the association between implant location and feature importance by the high variance we observed in gamma band power effect size and direction. When we examine the magnitude of the gamma band power effect in different implantation depths - mesial temporal, neocortical, and both - we see a stronger, but insignificantly different, effect size in neocortical patients than the other groups (**Figure S7A**). Most noticeably the effect is smallest in the “both” implant location patients. After accounting for covariance between the features as well as spike rate using a multivariate machine learning model we similarly sought to see the relationship between coefficient direction/magnitude and implant location in our PvT models (**Figure 7B**). While none of the coefficient distributions were significantly different between mesial temporal and neocortical implants, we do observe some general trends. First, we see a stronger association between dIEA amplitude and connectivity features in mesial temporal implants. Second, we see a negative median association between dIEA amplitude and gamma band power in neocortical patients while we see a positive median association in mesial temporal patients. This suggests that the high variance in directionality that we observe in the univariate analysis could be associated with implant location, but we are underpowered to verify this relationship statistically.

### Alternative parameter analyses

We sought to determine the robustness of our findings to the assumptions and parameters we selected in our methods. Thus, we varied two parameters– our choice of periodogram peak, and our method of combining feature values across channels– in two alternative analyses.

First, in the main manuscript, we investigated multidien cycles derived from the strongest LE phase-locked peak instead of the most prominent peak, as we felt that exploring the cycle most related to seizure occurrence was more meaningful. However, previous work has explored cycles of the most prominent peak, so we felt a comparison was warranted. We repeated our main analysis using cycles associated with the most prominent peak instead of the most phase-locked peak. Interestingly we found that there was less of a difference in model performance between scheduled events and post-stimulation events (**Figure S11**), and that model performance was lower overall at the group level. PvT scheduled event median AUC was 0.61 [0.53, 0.69], and the post-stimulation median AUC 0.58 [0.54, 0.64]. Thus the same features were better able to predict peak vs. trough of LE phase-locked cycles more than cycles related to the most prominent peak.

Second, in our original work, due to our limited number of data samples, we sought to reduce the dimensionality of our feature space by using a single statistic to summarize features across the four RNS channels. We used the maximum feature value across channels to achieve a similar amount of feature variance across patients, such that a patient with one active, high-variance channel and three low-variance channels could be comparable to a patient with four high-variance channels. However, using the mean feature value across channels is an important alternative method to consider, as it would consistently include contributions from all channels. Thus we repeated our analysis and we found that choosing the average versus the maximum feature value across channels as our summary statistic did not have a great impact on our results – Peak-Trough models still out-performed Rising-Falling models (*p* < 0.05), and post-stimulation model performance was reduced compared with SE models with the PvT scheduled event median AUC at 0.64 [0.56, 0.69] and the post-stimulation median AUC 0.59 [0.55, 0.65] (**Figure S11**).

###

### Alternative and multivariate classifiers

We explored alternative phase bin comparisons to better understand how spectral and connectivity features could discriminate between adjacent phase quadrants. We specifically built two models comparing the peak vs. the rising phase (PvR) and the rising vs the trough phase (RvT) because of reported seizure entrainment to the rising phase of dIEA cycles (Baud et al., 2021). The two models were trained following the same protocol as the protocol described in the methods section. We observed that there was a better ability to distinguish between the peak vs the rising (10/19 significant) phase than rising vs trough (6/19 significant) phases (permutation test, n = 1000, p < 0.05) (**Figure S10A**), but the difference between the two AUC distributions was not significant. When examining the feature distributions of the two models, we see that a number of feature distributions were significantly higher at the peaks than the rising phase (**Figure S10B**). However, we observed no significant feature distributions in our RvT models. The insignificant coefficient distributions combined with fewer significant models across the patient population suggests an asymmetric relationship between the rising phase and the peaks/troughs.

Additional biomarkers for understanding multidien cycles in seizure risk presents the potential for great clinical benefit. To this effect, we developed a four-class classifier to determine how well spectral power and connectivity could discriminate between all four phase bins continuously. The classifiers were trained following the same protocol as our binary models using matlab’s *fitecoc* function and a linear SVM kernel. We trained a multivariate model on each of the four feature sets that we examine throughout the paper: EEG features, EEG features + spikes, spikes, and post-stim EEG features. We observed similar trends in model performance to our PvT binary models with 9/19 patients having a significant EEG features model, 12/19 EEG features + spikes, 0/19 spikes, and 10/19 post-stim EEG features (permutation test, n=1000, p < 0.05). The descriptive statistics of the EEG feature models suggest that the patient-specific models are highly specific with the exception of the trough class, which could be in part due to sample class balance. We also observe significantly lower accuracy distributions in the spikes only models compared to EEG and EEG + spikes (**Figure S10C**).

## References

[1] P. J. Karoly *et al.*, “Multiday cycles of heart rate modulate seizure likelihood at daily, weekly and monthly timescales: An observational cohort study,” Neurology, preprint, Nov. 2020. doi: 10.1101/2020.11.24.20237990.

[2] C. Torrence and G. P. Compo, “A practical guide to wavelet analysis,” *Bull. Am. Meteorol. Soc.*, vol. 79, no. 1, pp. 61–78, Jan. 1998, doi: 10.1175/1520-0477(1998)079<0061:APGTWA>2.0.CO;2.

[3] M. Farge, “Wavelet transforms and their applications to turbulence,” *Annu. Rev. Fluid Mech.*, vol. 24, pp. 359–457, 1992.

[4] M. O. Baud *et al.*, “Multi-day rhythms modulate seizure risk in epilepsy,” *Nat. Commun.*, vol. 9, no. 1, p. 88, Dec. 2018, doi: 10.1038/s41467-017-02577-y.

[5] P. J. Karoly *et al.*, “Circadian and circaseptan rhythms in human epilepsy: a retrospective cohort study,” *Lancet Neurol.*, vol. 17, no. 11, pp. 977–985, Nov. 2018, doi: 10.1016/S1474-4422(18)30274-6.

[6] D. N. Anderson *et al.*, “Closed-loop neurostimulation for epilepsy leads to improved outcomes when stimulation episodes are delivered during periods with less epileptiform activity,” Neurology, preprint, Nov. 2022. doi: 10.1101/2022.11.28.22282784.

[7] C. Torrence and G. P. Compo, “A Practical Guide to Wavelet Analysis,” *Bull. Am. Meteorol. Soc.*, vol. 79, no. 1, pp. 61–78, Jan. 1998, doi: 10.1175/1520-0477(1998)079<0061:APGTWA>2.0.CO;2.

[8] W. F. Massy, “Principal Components Regression in Exploratory Statistical Research,” *J. Am. Stat. Assoc.*, vol. 60, no. 309, pp. 234–256, Mar. 1965, doi: 10.1080/01621459.1965.10480787.

[9] B. Phipson and G. K. Smyth, “Permutation P-values should never be zero: calculating exact P-values when permutations are randomly drawn,” *Stat. Appl. Genet. Mol. Biol.*, vol. 9, p. Article39, 2010, doi: 10.2202/1544-6115.1585.

[10] P. Berens, “CircStat: A MATLAB toolbox for circular statistics,” *J. Stat. Softw.*, vol. 31, pp. 1–21, Sep. 2009, doi: 10.18637/jss.v031.i10.

## Supplementary Figures


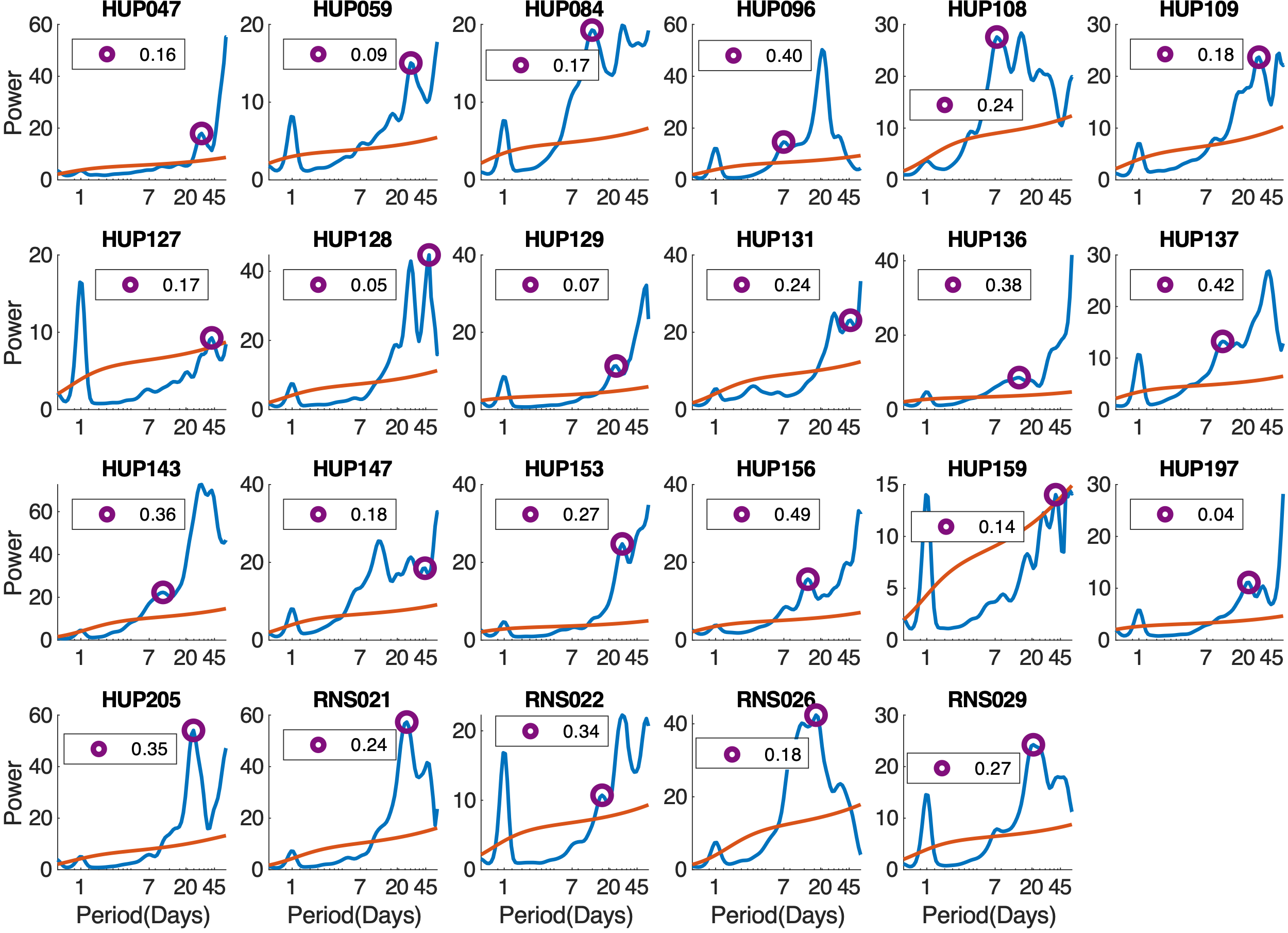


**Figure S1. Individual dIEA count periodograms**. Periodograms showing the average power over time for a range of dIEA cycle periodicities. The red line denotes the 99% confidence interval of the red noise null model described in *Torrence and Compo et. al., 1998*. We fit the null model using first order autoregressive coefficients of the longest continuous segment of dIEA detections after normalizing between clinician visits. The purple circle marks the multidien peak that has the highest phase entrainment of Long Episodes among all multidien peaks, with the actual phase locking value of Long Episodes to cycle phase in the legend. Plots show inter-patient variability in terms of the prominence of circadian cycles as well as the period length of dIEA count dominant modes.


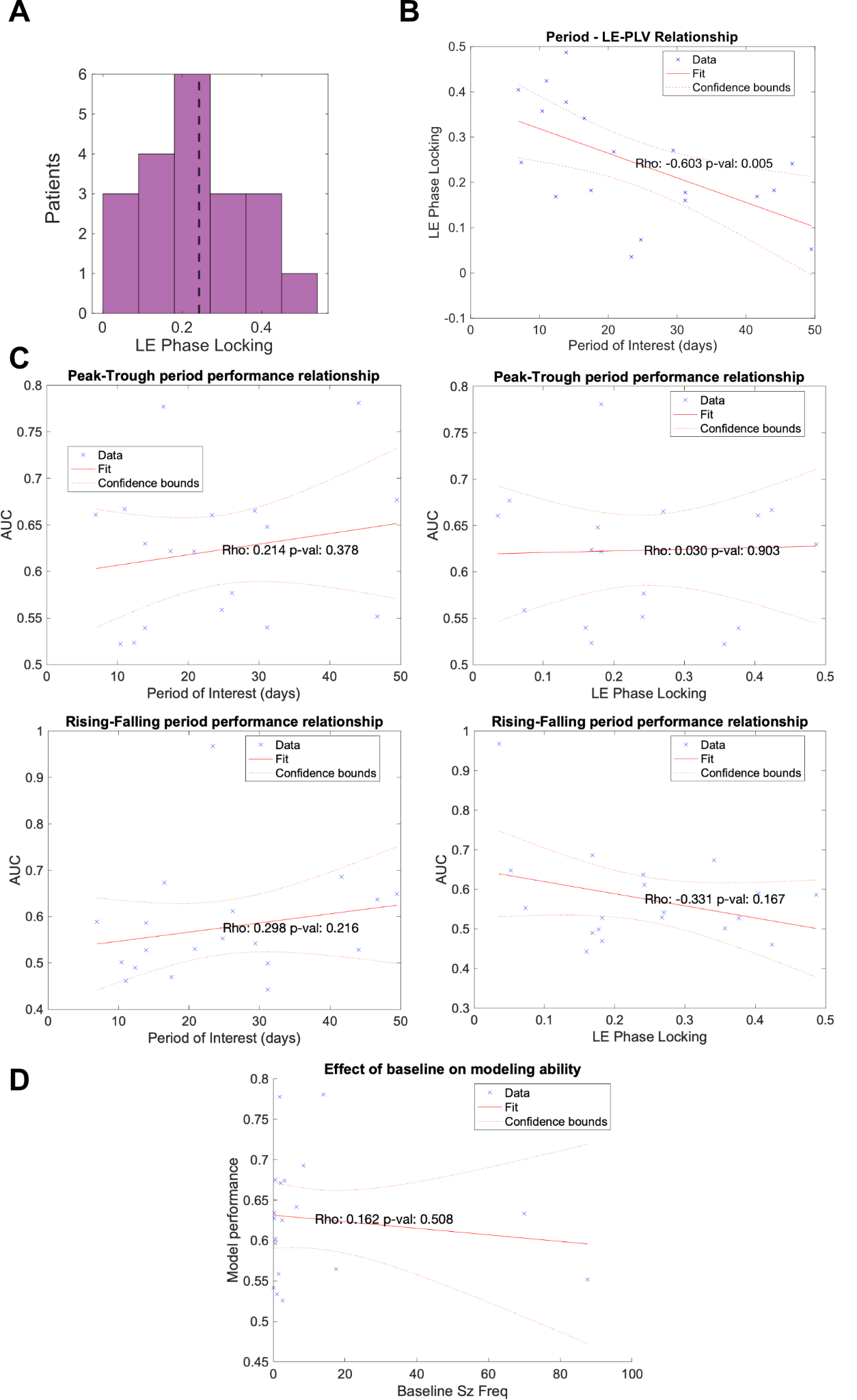


**Figure S2. dIEA feature correlations. A)** Distribution across all patients of LE phase locking value. Each sample in the distribution is the highest LE PLV across all dominant modes of the dIEA signal for each patient. LE PLV was calculated by taking the axial mean of the LE distribution across cycle phase (**Figure 1C**) using the circ-stat toolbox [10]. **B)** Spearman rank correlation between the magnitude of LE phase locking and the period of the associated cycle. **C)** Spearman rank correlation between PvT and RvT model AUC and LE phase locking at the period of interest and the length of the period of interest. **D)** Spearman rank correlation between PvT model performance and baseline seizure frequency measured in seizures per week.


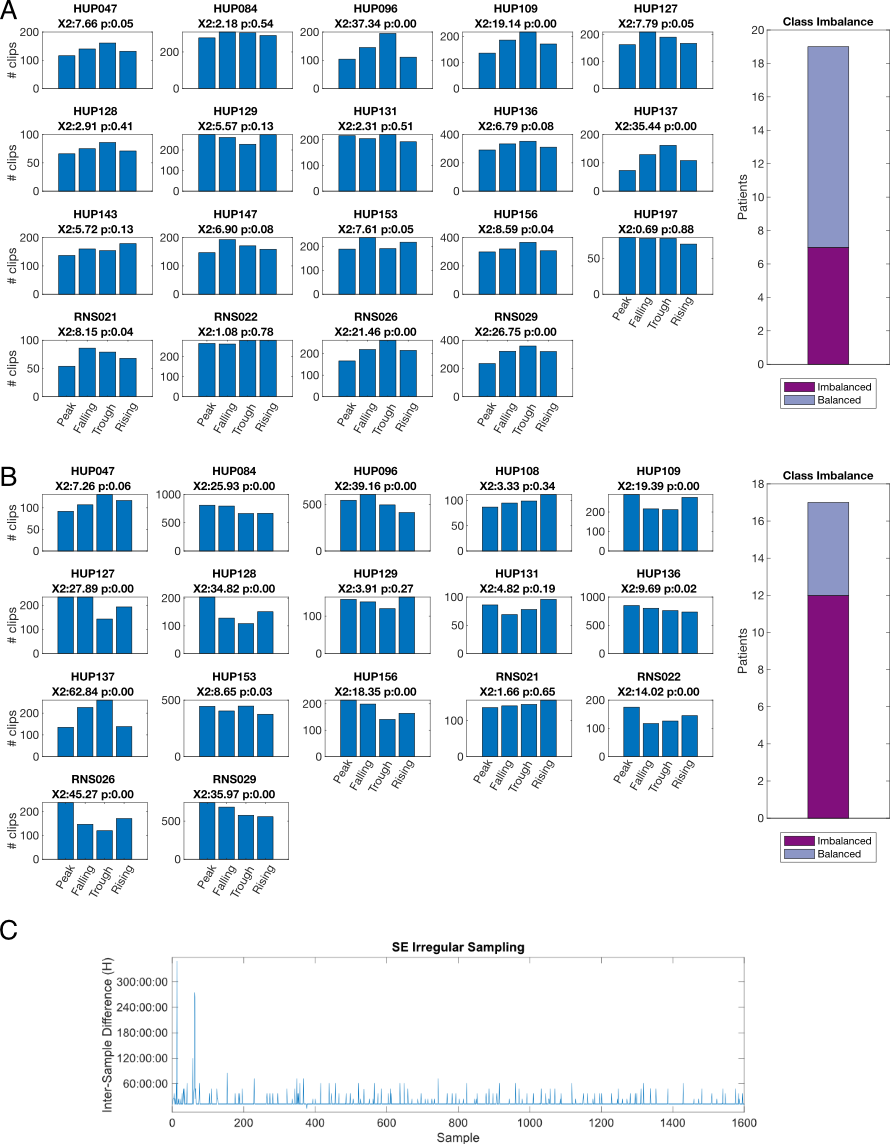


**Figure S3. Patient-specific sample distributions. (A) scheduled events without stimulation, (B) scheduled events with stimulation.** Each subplot represents the distribution of recordings with (B) and without (A) stimulation across each of the four cycle phase categories. The bar plot to the right of each population plot shows the number of patients with a significantly imbalanced class distribution (p < 0.05). Chi-squared test statistic and p value up to two decimal points shown in figure title. p: 0.00 is equivalent to p < 0.01. **C)** Plot showing the inter-sample interval in hours between each scheduled event for an example patient (HUP096).


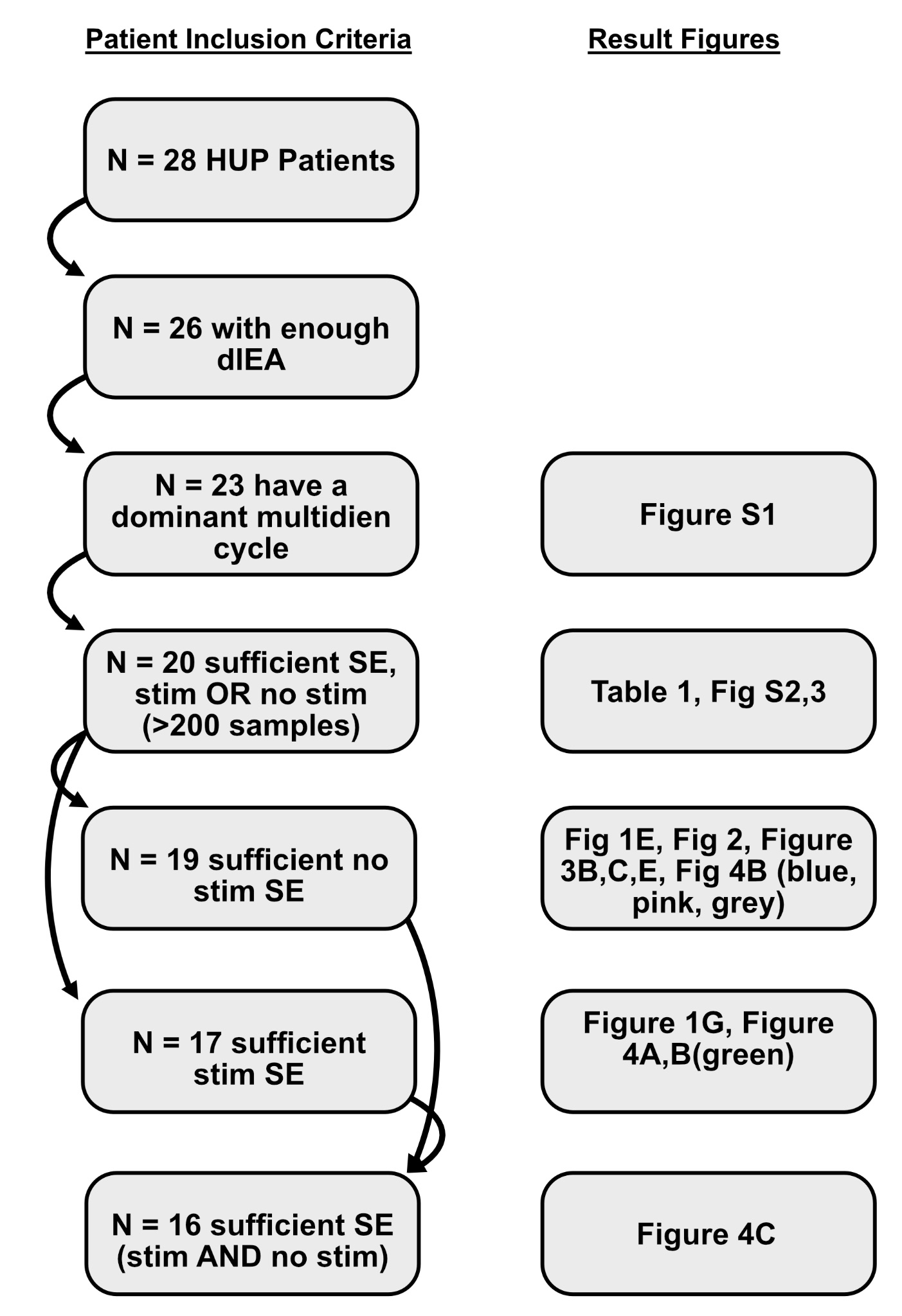


**Figure S4. Patient population flowchart.** Diagram showing patient exclusion flow chart (left) as well as which figures each patient population is included in (right). Colors refer to boxplot containing the associated patient population displayed at left.

**
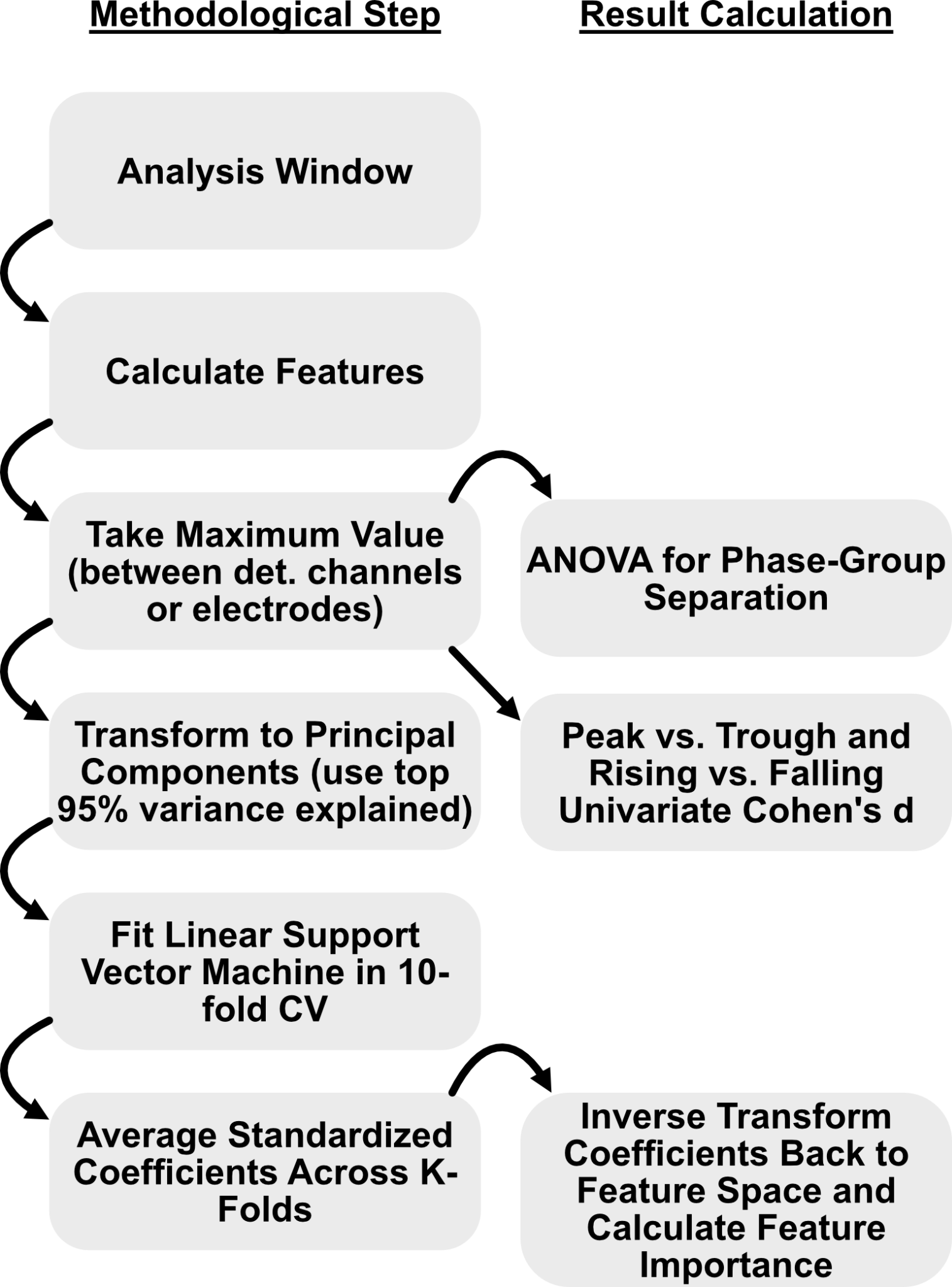
**

**Figure S5. Feature analysis flowchart.** Analysis pipeline for both univariate and multivariate analyses in both non-stimulation SEs and post-stimulation windows. Flowchart shows the order of steps for calculating feature importance in two manners: univariate effect size, and standardized model coefficient. For multivariate analysis, we use the minimum required number of components to explain 95% of the variance in the data to remove principal components associated with spurious correlation between the features [8]).


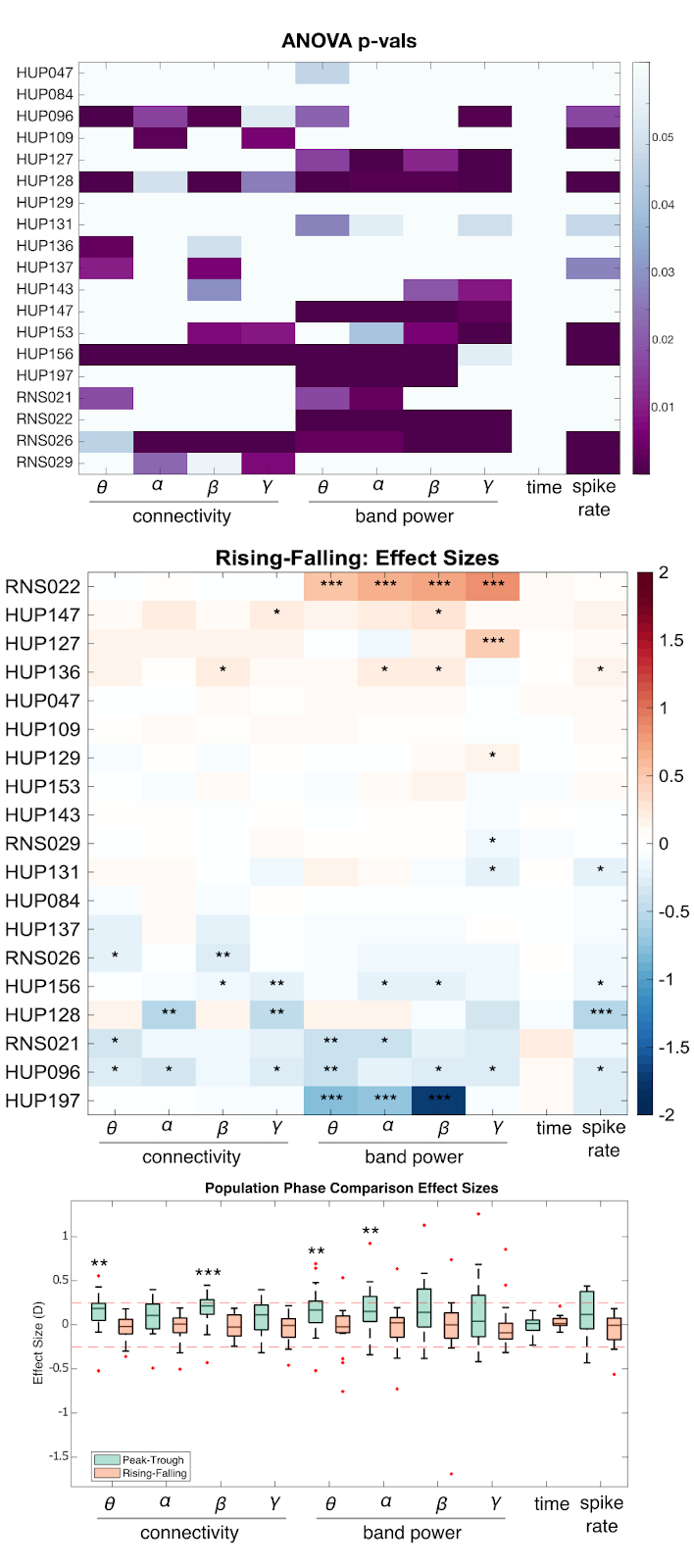


**Figure S6. Additional univariate analyses.** A) One-way ANOVA for phase group separation. For each feature and patient we performed an anova to determine if each feature could discriminate at least one phase group from the rest. B) Rising-Falling effect sizes. We also calculated the difference in each feature distribution between the rising and falling phases of the cycles (peak-trough shown in **Figure 3B**). Significance of the separation in features between phase groups is denoted in stars (* p < 0.05, ** p < 0.01, *** p < 0.005). C) We lastly show the population distribution of effect sizes for each feature. Red-dashed lines indicate lower bound for moderate effect size (± 0.25). Stars indicate significance level of effect size distributions against a zero median (signtest).


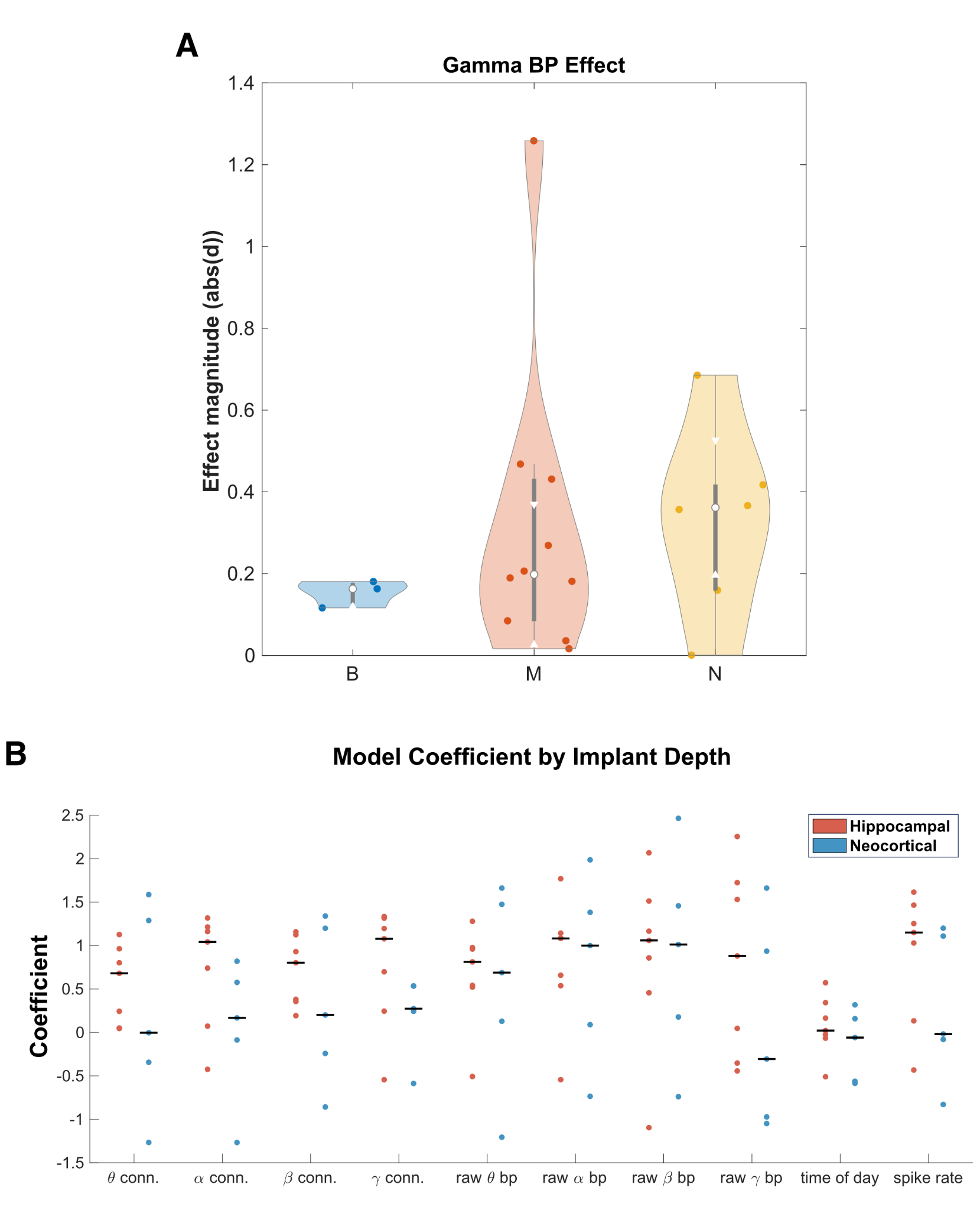


**Figure S7. Feature importance by implant depth.** A) Magnitude of gamma band power effect size grouped by patients with neo-cortical foci (N), mesial temporal foci (M) or both (B). B) PvT model coefficients of patients with significant PvT models separated by implant depth. No feature significantly differed between patient groups (Mann-Whitney U, p > 0.05). A positive coefficient value signifies a higher feature value at the peaks of the dIEA cycle.

**
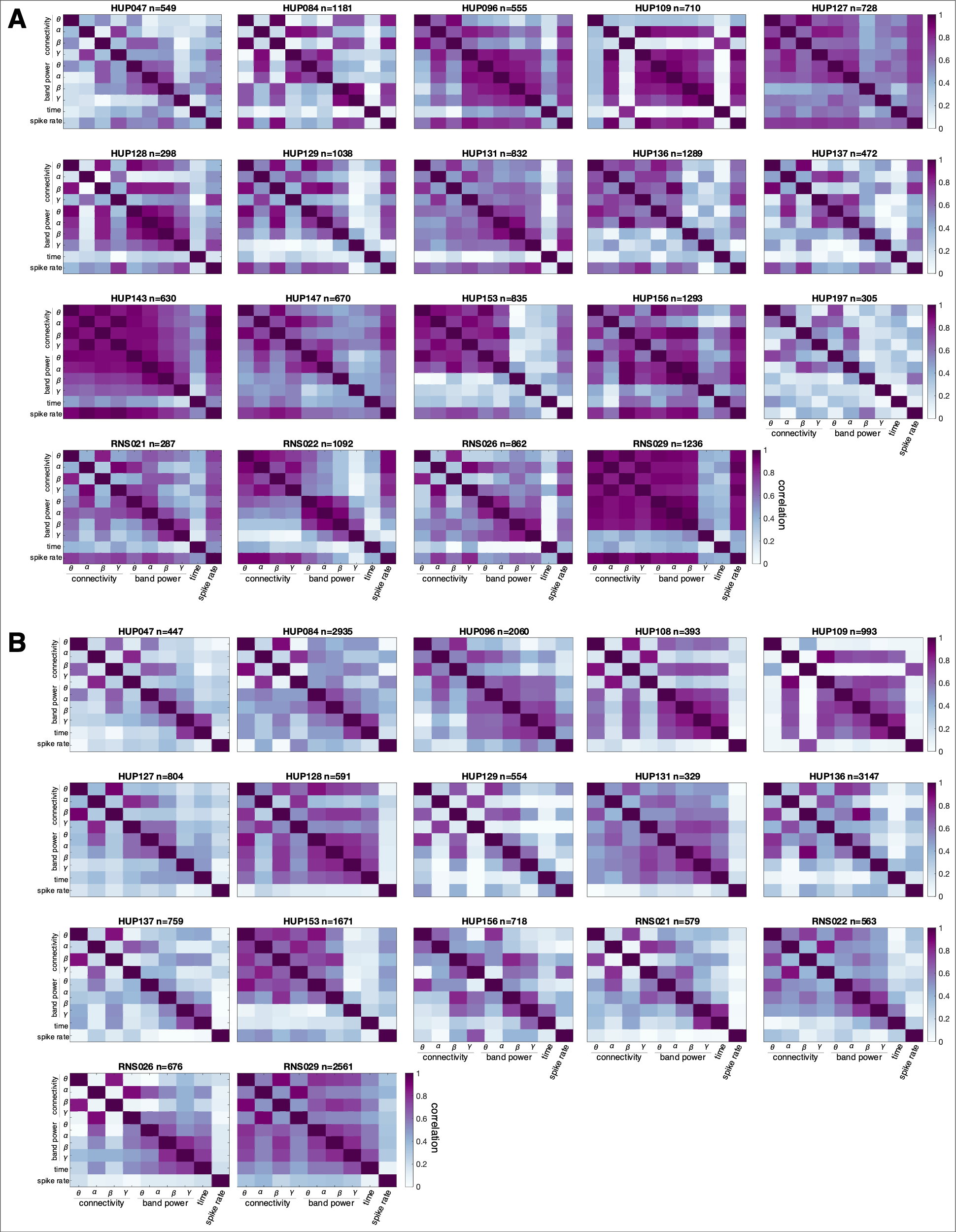
**

**Figure S8. Feature correlation.** Features from scheduled events without stimulation (A), and with stimulation (B). Qualitative group level analyses reveal that there is high multicollinearity between features in both the stimulation and no-stimulation groups, but which features are correlated is not necessarily consistent across patients.


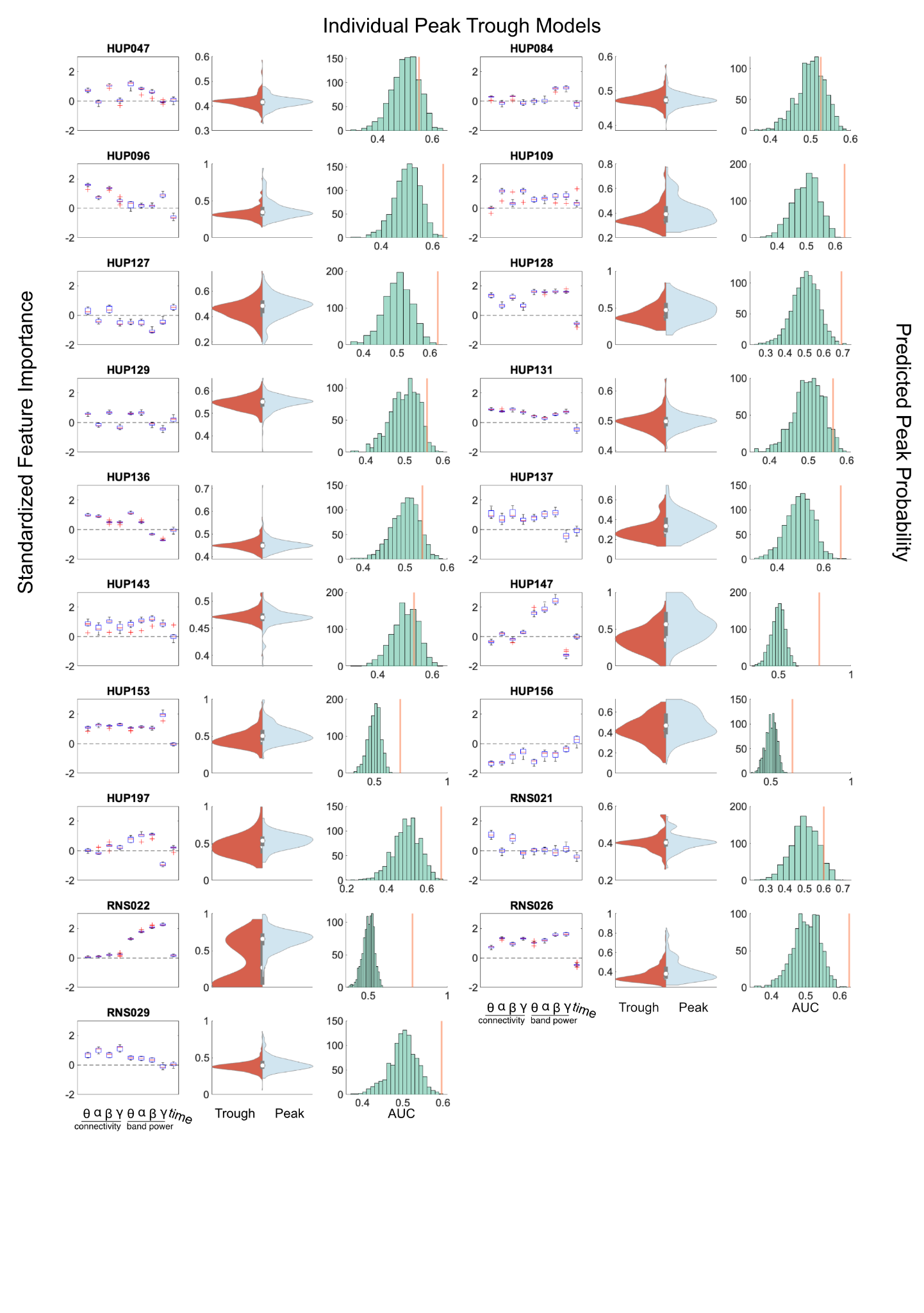


**Figure S9. Background EEG Model performances.** Patient level feature maps showing the distributions of linear SVM model coefficients across k-folds for peak-trough models. [0,1] scaled predicted probability distributions to the right of each feature distribution shows class separation with the true peak on the right and true trough samples on the left. The AUC and p-value associated with out of sample model performance for both the peak-trough (shown) and rising-falling models is shown in the patient information table. Histograms to the right of the predicted probability distributions show the null AUC distribution in green, with the true AUC value represented as the orange vertical line.


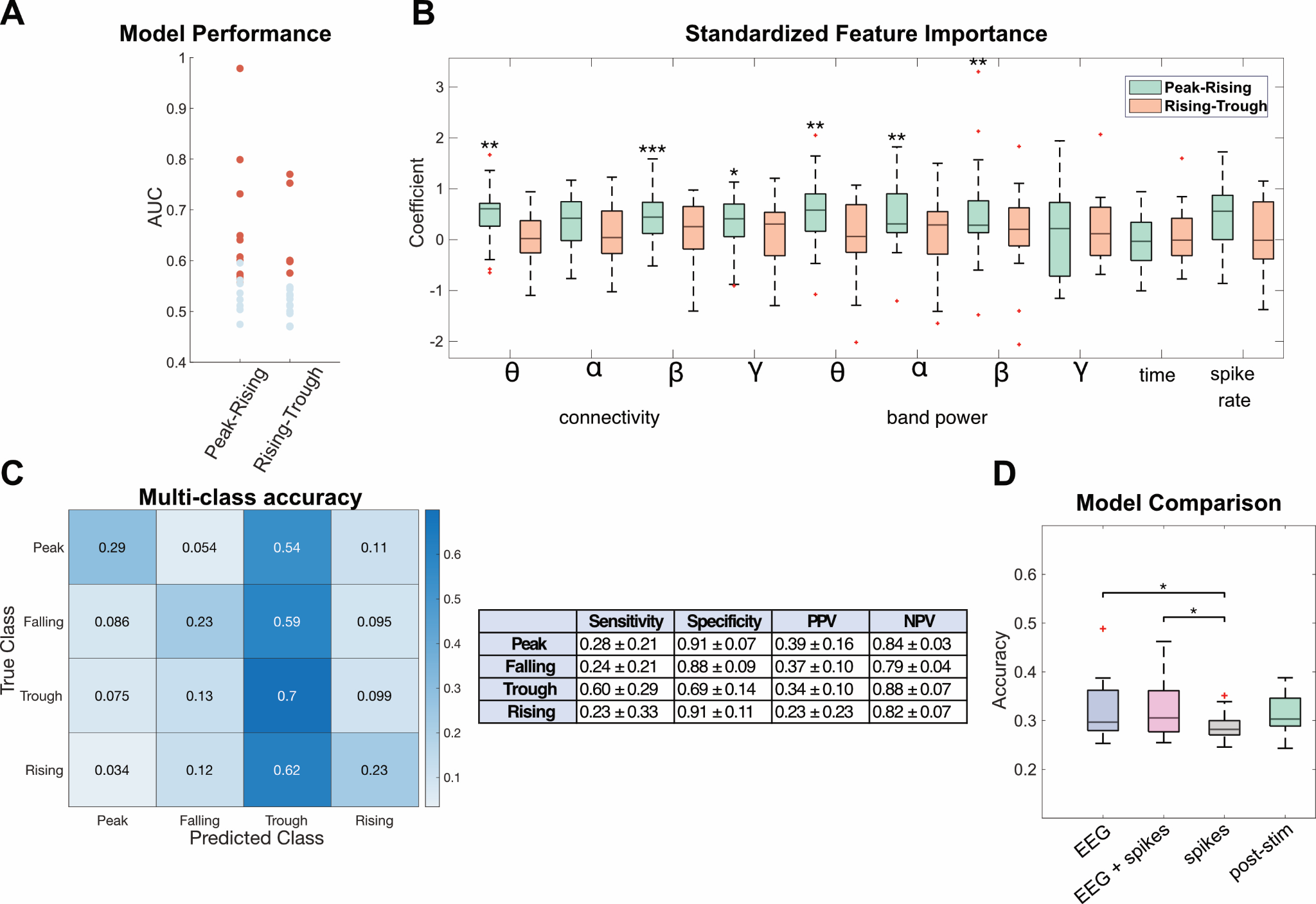


**Figure S10. Alternative classifiers.** A) Distribution of peak vs rising (PvR) and rising vs trough (PvT) models with statistically significant models (one-sided permutation test, n=1000, p < 0.05) in orange and insignificant models in blue. B) Standardized coefficient distributions of the significant PvR (green) and RvT(orange) models. Star signifies significance and a higher value means the feature is higher at the dIEA cycle peak(rising) phase. C) Relative confusion matrix for a four-phase classifier with EEG features averaged across all patients with a significant model (permutation test, p < 0.05). Table shows the sensitivity, specificity, and positive and negative predictive values (mean +/- std) generated from the confusion matrix. D) Accuracy comparison across four different feature sets: EEG, EEG + spikes, spikes only, and post-stim EEG features. star signifies pairwise significance (p < 0.05).


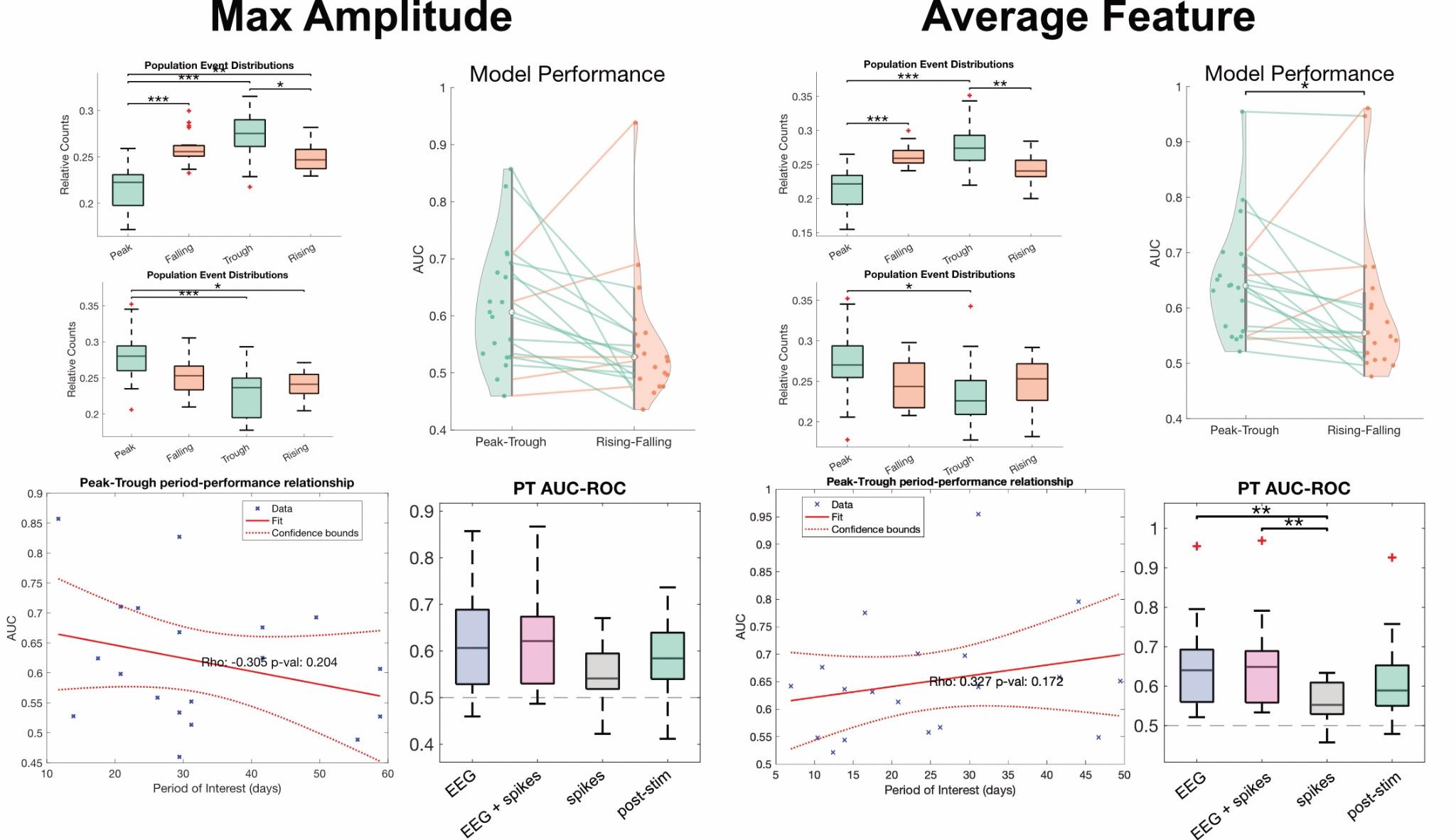


**Figure S11. Model parameter sensitivity analysis.** Here we show the adjusted results using both the maximum amplitude dIEA cycle (left), and the average feature value between the two detection features rather than the maximum (right). For each alternative model parameter, we show the phase distribution of SEs for both stim and no-stim scenarios (top left), the distribution of AUC values between PvT and RvF models (top right), the PvT period-performance correlation (bottom left), and the AUC distribution comparison across feature sets.


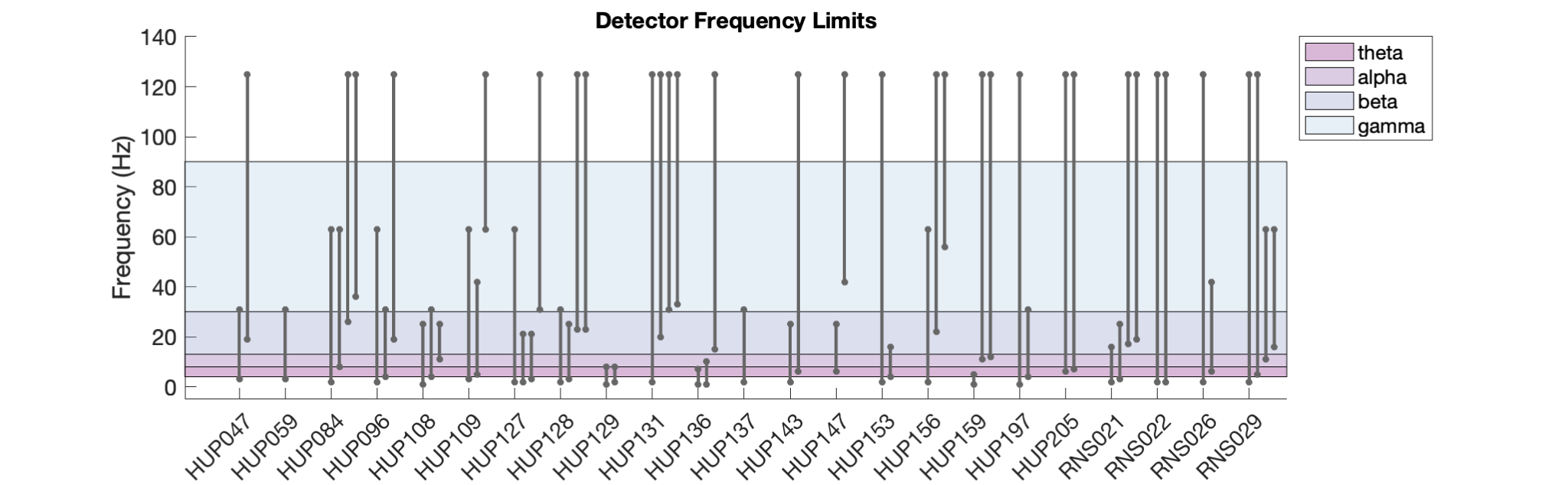


**Figure S12. Detection parameter frequency ranges.** Vertical bars span the frequency range for a given band power threshold detector. Patients may have up to four detection settings at one time, with different frequency ranges, and threshold levels. Detection parameters can change over time, so the frequency intervals represented here are a snapshot of the detection settings that remained unchanged for the longest time span within a patient’s dataset.

**Supplemental Tables**

**Table S1.  Patient-level multivariate model performance**

| **ID** | **PT AUC** | **PT p-val** | **RF AUC** | **RF p-val** |
| --- | --- | --- | --- | --- |
| HUP047 | 0.55 | 1.54E-01 | 0.44 | 8.91E-01 |
| HUP084 | 0.53 | 2.53E-01 | 0.49 | 7.02E-01 |
| HUP096 | 0.64 | **9.99E-04** | 0.59 | **2.10E-02** |
| HUP109 | 0.63 | **9.99E-04** | 0.48 | 6.93E-01 |
| HUP127 | 0.62 | **9.99E-04** | 0.69 | **9.99E-04** |
| HUP128 | 0.69 | **3.00E-03** | 0.65 | **1.00E-02** |
| HUP129 | 0.56 | **3.20E-02** | 0.55 | 1.02E-01 |
| HUP131 | 0.56 | **4.20E-02** | 0.63 | **9.99E-04** |
| HUP136 | 0.54 | 9.29E-02 | 0.53 | 2.21E-01 |
| HUP137 | 0.67 | **2.00E-03** | 0.48 | 6.83E-01 |
| HUP143 | 0.53 | 2.61E-01 | 0.51 | 4.64E-01 |
| HUP147 | 0.78 | **9.99E-04** | 0.51 | 4.50E-01 |
| HUP153 | 0.67 | **9.99E-04** | 0.57 | **4.00E-02** |
| HUP156 | 0.63 | **9.99E-04** | 0.59 | **3.00E-03** |
| HUP197 | 0.67 | **9.99E-04** | 0.96 | **9.99E-04** |
| RNS021 | 0.60 | 7.49E-02 | 0.60 | 5.89E-02 |
| RNS022 | 0.78 | **9.99E-04** | 0.67 | **9.99E-04** |
| RNS026 | 0.63 | **9.99E-04** | 0.46 | 8.02E-01 |
| RNS029 | 0.60 | **9.99E-04** | 0.53 | 1.95E-01 |
